# Classification and conservation priority of five Deccani sheep ecotypes of Maharashtra, India

**DOI:** 10.1371/journal.pone.0184691

**Published:** 2017-09-14

**Authors:** Dinesh Kumar Yadav, Reena Arora, Anand Jain

**Affiliations:** ICAR-National Bureau of Animal Genetic Resources, Karnal (Haryana), India; Estonian Biocentre, ESTONIA

## Abstract

Characterization of Indian livestock breeds has mostly been limited to single breed/population focused on either physical description of traditionally recognized breeds/populations or to their genetic description. Usually, morphological and genetic characterization has taken place in isolation. A parallel morphological characterization of genetically identified breeds or genetic characterization of morphologically described breeds is mostly missing, and their conservation priorities have largely been based on solely considering degree of endangerment. This study uses parallel approach based on morphometric and genetic differentiation for classification of five sheep ecotypes of Maharashtra state, and sets their conservation priority using threat parameters, current utilities/merits and contribution to genetic diversity. A total of 1101 animals were described for 7 body measurements for morphometric characterization. From this sample set, 456 animals were genotyped for 25 microsatellite markers for genetic characterization. Conservation priorities were assessed combining genetic and non-genetic factors. All studied traits varied significantly among ecotypes (p<0.05). All morphometric traits exhibited substantial sexual dimorphism except ear length. Males were 42% heavier than females. Madgyal sheep were the largest amongst the five ecotypes. In the stepwise discriminant analysis, all measured traits were significant and were found to have potential discriminatory power. Tail length was the most discriminatory trait. The Mahalanobis distance of the morphological traits between Kolhapuri and Madgyal was maximum (12.07) while the least differentiation was observed between Madgyal and Solapuri (1.50). Discriminant analysis showed that 68.12% sheep were classified into their source population. The Sangamneri sheep showed least assignment error (22%) whilst Solapuri exhibited maximum error level (41%). A total of 407 alleles were observed, with an average of 16.28 alleles per locus. Sufficient levels of genetic diversity were observed in all the ecotypes with observed heterozygosity values exceeding 0.47 and gene diversity values exceeding 0.76. About 6% of the total genetic variation was explained by population differences (F_ST_ = 0.059). Pairwise F_ST_ values indicated least differentiation between Solapuri and Madgyal (0.025). In terms of genetic distances, Kolhapuri and Lonand were most closely related (Ds = 0.177). The most probable structure clustering of the five studied populations was at *K* = 5. The study showed a fair congruence between the dendrogram constructed on the basis of Mahalanobis distances and Nei’s as well as Reynolds genetic distances. The findings gave highest conservation priority to Lonand and least to Solapuri ecotype.

## Introduction

The Global Action Plan for Animal Genetic Resources recognizes that “a better understanding of the characteristics of livestock breeds is necessary for guiding decision making in the development of farms and breeding programs”[1]. Genetic and phenotypic characterization are the most powerful tools which define the breed standards in farm animal genetic resources. Sheep biodiversity has been described using morphological measurements [2–7]. Several authors have used the analysis of morphological traits for differentiating populations and breeds [8–12]. In addition, DNA based microsatellite studies have been used successfully to characterize the sheep genetic diversity [8, 13–15], though a parallel approach is an exception rather than a practice. The phenotypic description remains insufficient lest should be supported by multivariate statistical analysis of morphometric traits. The description also solicits the genetic characterization support in order to design pragmatic conservation and utilization strategies/programs.

Indian sheep breeds have generally been named after their place of origin and some based on their prominent characteristics. These have been traditionally described and classified based on their utility, and on the basis of agro-ecological regions [16]. In recent past, the ovine characterization program has focused mostly on single breed/population. The morphometric and genetic characterization has taken place in isolation. Though, genetic variation and relationships across majority of currently recognized sheep breeds have been described [13, 17–18]. Yet, morphological and genetic structuring patterns have not been investigated fully due to lack of parallel approach of genetic and morphometric characterization. Conservation decisions can be based on a number of different considerations, with the degree of endangerment being the most important [19]. Limited resources force to concentrate efforts on only a few breeds under threat. We need insight into genetic variations present in each breed. Weitzman proposed a method to quantify the diversity in a set of populations [20]. Ruane proposed a conservation framework that incorporates both genetic diversity and breed merits for prioritizing breeds at the national level [21]. In India, applications of this framework are lacking and conservation priorities have largely been based on solely considering degree of endangerment.

The sheep of Deccan plateau, between 16° to 20°N latitude and 72° to 78°E longitude, called Deccani were considered a breed [22–23] and were defined as a medium sized sheep, predominantly black or black with white markings; white and brown/fawn animals are also seen. Some authors [23–24] reported strains/subpopulations of Deccani in Maharashtra on the basis of different coat colour. Owing to their locations these were called Lonand, Sangamneri, Solapuri, Madgyal and Kolhapuri. A preliminary study [25] described them morphologically. Nevertheless, sheep ecotypes or geographically separated populations do exist in India and several such populations are spread over different regions of the country. The present study builds strength over the limitations of the characterization and conservation programs and emphasizes objective assessment of defining national breeds and their conservation priorities. Our results can be useful for classification of Indian sheep as well as other livestock and implementing their conservation plans based on their ranking on regional or national level.

## Materials and methods

### Sampling strategy

#### Study sites and sampling information

**Fig 1:**
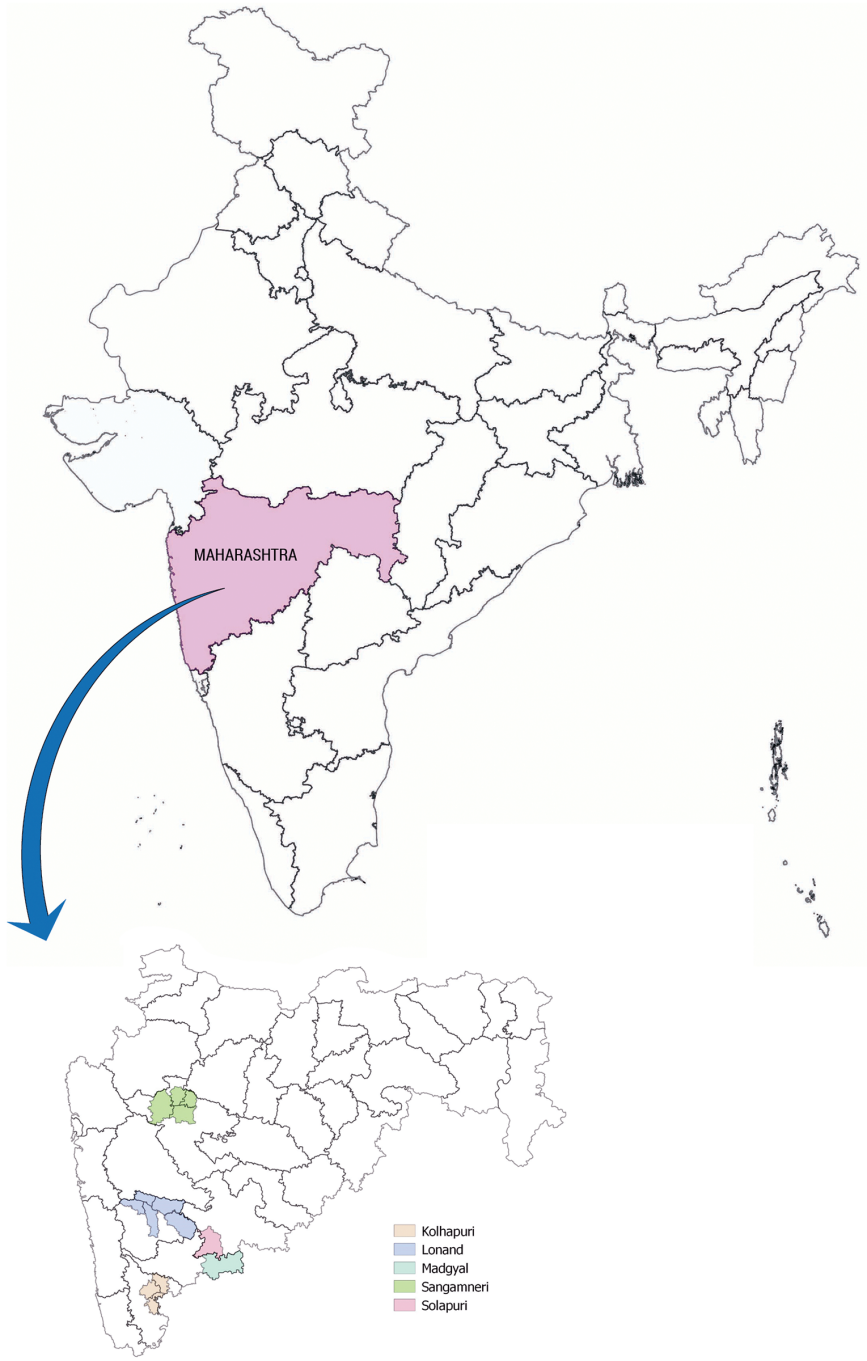
Distribution area of the five sheep ecotypes.

Maharashtra state (15°55' and 22° N and 72°5' and 80°9' E) is located in the Deccan plateau region of India. The altitude of the plateau varies from 450–750 m above mean sea level. The state is divided into 36 administrative districts grouped into 6 divisions. The studied sheep ecotypes are native to and distributed through five districts as shown in Fig 1. Purposive sampling was used to determine the distribution area based on the opinion of sheep development experts and the shepherds. The age was determined by dentition and the animals having two or more permanent teeth were included in the study. A dial spring balance of 100 kg × 500 gm. (capacity 100 kg; least count 500 gm.) was used to record live body weight in kilogram (kg). Holding the animal in normal standing position, body dimensions were measured using a steel tape of 5 m length of class II accuracy with records taken to the nearest centimetre (cm). Morphometric traits studied were body length (BL), height at withers (HW), chest girth (CG), paunch girth (PG), ear length (EL), tail length (TL) and live body weight (BW). The number of animals sampled in each flock ranged from 5 to 10, with morphometric measurements recorded for a total of 1101 animals; and for blood samples ranged from 2 to 4 on same set of animals, with 456 animals subjected to microsatellite genotyping. Supplementary S1 Table gives the ecotype wise number of animals used for morphometric characterization and genotyping. The animals were reared under extensive management system.

### Ethical statement

The study involved drawing of ~ 5 ml blood from jugular vein aseptically from sheep with the consent of the flock owners in the presence of the trained veterinarians. There is no specific legislation for blood sample collection and hence no approval was necessary. Nevertheless, the study has the approval of Institute Animal Ethics Committee (IAEC) of Indian Council of Agricultural Research –National Bureau of Animal Genetic Resources, Karnal (PME Ref. 11/2017-18).

### Genotyping

A total of 456 blood samples of unrelated animals were collected from five sheep ecotypes across their distribution tract. Samples were taken from distinct flocks. Number of samples per flock ranged between 2 to 4 depending upon the size of the flock. The sheep owners do not maintain any pedigree records of the animals.

Genomic DNA was isolated and purified using the standard phenol chloroform extraction protocol. Genetic variation was assayed as described earlier [13] using 25 microsatellite markers recommended for ovines [26–27]. Details of microsatellite markers used in the study are given as supplementary S2 Table. The genotyping was carried out on an ABI 3100 automated DNA sequencer using LIZ 500 as the internal size standard.

### Statistical analysis

Statistical analyses on morphometric data were performed using SAS software, version 9.3 [28]. The analysis of variance was performed using the Mixed procedure fitting a model that included the random effect of animal and the fixed effects of gender and age of animal. A canonical discriminant analysis was performed, using Candisc procedure, for determining morphometric traits most discriminating the populations. Then, the probabilities of including an individual in a population were determined using Discrim procedure based on the linear discriminant function that included the seven morphometric variables. Mahalanobis distances [29] generated during the canonical discriminant analysis were used to construct a dendrogram using the Unweighted Pairs Group Method Analysis.

Allele sizing was performed using GENEMAPPER software. Allele frequencies, observed number of alleles (N_a_), observed heterozygosity (H_o_), expected heterozygosity (H_e_), F_IT_ (total inbreeding estimate), F_ST_ (measurement of population differentiation) and F_IS_ (within-population-inbreeding estimate) were calculated using FSTAT ver 2.9.3 [30]. Polymorphism information content (PIC) was calculated according to [31]. Exact tests for deviations from Hardy-Weinberg equilibrium for all locus-population combinations and linkage disequilibrium between pairs of loci were assessed by GENEPOP [32] version 3.1. 1. The program estimated the exact p-values using Markov chain (dememorization 10000, batches 20, iteration per batch 5000). BOTTLENECK (version 1.2. 02) software [33] was used for detection of bottleneck effect if any, in the investigated sheep. All the F statistics were computed using 1000 permutations. Microsatellite allele frequency data was applied to calculate genetic distances by employing Nei’s [34] original measures incorporated in PopGen32 [35]. Nei’s (1972) [34] and Reynolds (1983) [36] original distance measures between the 5 ecotypes were then used for tree construction based on UPGMA method using POPULATIONS 1.2.28 [37] software. Ganjam sheep (40 samples) from the eastern region of India were taken as out-group. Due to unequal sampling across the ecotypes, 50 samples from each ecotype were used to estimate the genetic distances. Bootstraps of 1000 replicates were performed in order to test the robustness of tree topology. Principal Component Analysis was performed using GenAlex program [38]. Population assignment was performed as per [39]. Structure version 2.3.1 [40] was used to analyse the genetic structure of the populations using the Bayesian Markov chain Monte Carlo approach. Nine different runs from K = 1 to K = 9 were carried out to identify the most likely number of clusters present in the dataset. Ten independent runs were performed for each K. The analysis was performed by means of the admixture model with correlated allele frequencies with a burn in period of 100000 and 100000 iterations for data collection.

### Conservation priority

Contribution to between-breed diversity was computed by estimation of Weitzman values [20] based on Nei’s genetic distances [34] with WEITZPRO [41] and the marginal loss of diversity attached to each ecotype was quantified. Threat status was assessed using FAO recommended indicators and extinction probabilities were estimated using [42]. Similarly, ranking of breeds according to their merits or current utility was done based on conceptual framework of [21]. The total utility of the i^th^ ecotype was estimated using conceptual framework of [43–44] as U_i_ = 2(z_i_+D_i_) +W_i_, where z_i_ is the extinction probability, D_i_ is Weitzman marginal diversity and W_i_ is the current merit of the i^th^ ecotype.

## Results

Least-squares means and standard errors for morphometric traits are given in Table 1. All traits varied significantly among ecotypes (p<0.05). Madgyal animals had the highest body measurements amongst the five ecotypes. Except EL and TL, all morphometric traits increased significantly with the age of animal (P < 0.05). All morphometric traits exhibited substantial sexual dimorphism except EL. Males were 42% heavier than females whereas in rest of the traits difference was 8–10%.

**Table 1:** Least square means (±SE) of morphometric traits of five sheep ecotypes^1^^,^ *^,^ **^,^ ***.

Fixed effects	BL	HW	CG	PG	EL	TL	BW
**Gender**	***	***	***	***	ns	***	***
*Female*	73.6±0.11^b^	72.2±0.11^b^	77.0±0.14^b^	76.9±0.15^b^	18.1±0.08^a^	17.4±0.07^b^	34.4±0.22^b^
*Male*	81.1±0.24^a^	79.1±0.24^a^	83.8±0.30^a^	83.24±0.33^a^	18.2±0.17^a^	19.1±0.15^a^	48.9±0.47^b^
*Sexual dimorphism*	1.10	1.10	1.09	1.08	1.00	1.10	1.42
**Age**	***	***	***	***	ns	ns	***
*2T*	76.1±0.24^b^	74.6±0.24^b^	77.4±0.31^c^	77.5±0.33^c^	18.1±0.18^a^	18.2±0.15^a^	38.1±0.48^c^
*4T*	77.6±0.22^a^	75.9±0.22^a^	80.2±0.27^b^	79.8±0.30^b^	18.3±0.16^a^	18.2±0.13^a^	41.6±0.43^b^
*6T*	78.1±0.24^a^	76.3±0.24^a^	82.2±0.30^a^	81.8±0.33^a^	18.3±0.18^a^	18.3±0.15^a^	44.3±0.48^a^
*8T*	77.6±0.17^a^	75.7±0.17^a^	81.9±0.21^a^	81.3±0.23^a^	17.9±0.12^a^	18.2±0.10^a^	43.8±0.33^a^
**Ecotype**	***	***	***	***	***	***	***
*Lonand*	75.8±0.29^d^	73.1±0.29^d^	77.7±0.37^c^	77.9±0.40^c^	15.1±0.22^d^	16.4±0.18^d^	36.0±0.58^d^
*Solapuri*	76.8±0.22^c^	77.1±0.22^b^	81.3±0.27^b^	81.1±0.30^b^	19.3±0.16^b^	18.9±0.14^c^	43.9±0.43^b^
*Madgyal*	80.7±0.21^a^	81.0±0.21^a^	84.4±0.26^a^	84.9±0.29^a^	20.2±0.15^a^	20.3±0.13^a^	50.9±0.41^a^
*Kolhapuri*	74.7±0.19^e^	71.7±0.19^e^	78.0±0.24^c^	76.0±0.26^d^	17.0±0.14^c^	15.9±0.12^d^	37.5±0.37^d^
*Sangamneri*	78.7±0.24^b^	75.2±0.24^c^	80.7±0.29^b^	80.9±0.33^b^	19.1±0.17^b^	19.7±0.15^b^	41.4±0.47^c^

^1^Levels not connected by same letter in a column are significantly different; BL, body length; HW, height at withers; CG, chest girth; PG, paunch girth; EL, ear length TL, tail length; BW, body weight; CV, coefficient of variation (%);

*Significance level: p<0.05;

** Significance level: p<0.01;

*** Significance level: p<0.0001;

Body weights are in kilogram, other traits are in centimetre

Results of stepwise discriminant analysis are presented in Table 2. Based on F-values and Wilk’s lambda, all the measured traits were significant (p<0.0001). All the variables in the data set were found to have potential discriminatory power. The Mahalanobis distances estimated between the five sheep ecotypes according to morphometric traits studied, are presented in Table 3. All pair wise distances were highly significant (*p* < 0.0001). The largest distance was between Kolhapuri and Madgyal (12.07) and the least was between Madgyal and Solapuri (1.50).

**Table 2:** Stepwise selection summary of traits.

Step	Trait entered	Partial R^2^	F-value	p>F	Wilk’s lambda	P<lambda	Average squared canonical correlation	p>ASCC
1	TL	0.467	240.95	***	0.533	***	0.117	***
2	HW	0.211	73.60	***	0.420	***	0.162	***
3	BL	0.308	122.17	***	0.291	***	0.225	***
4	EL	0.152	49.25	***	0.246	***	0.253	***
5	BW	0.076	22.53	***	0.228	***	0.269	***
6	PG	0.130	40.89	***	0.198	***	0.293	***
7	CG	0.094	28.50	***	0.179	***	0.307	***

*** p<0.0001

**Table 3:** Mahalanobis distance between the five sheep ecotypes.

**Ecotype**	Lonand	Solapuri	Madgyal	Kolhapuri	Sangamneri
Lonand	**0**	7.10	11.73	3.43	5.78
Solapuri		**0**	1.50	7.01	4.35
Madgyal			**0**	12.07	5.91
Kolhapuri				**0**	6.64
Sangamneri					**0**

**Fig 2:**
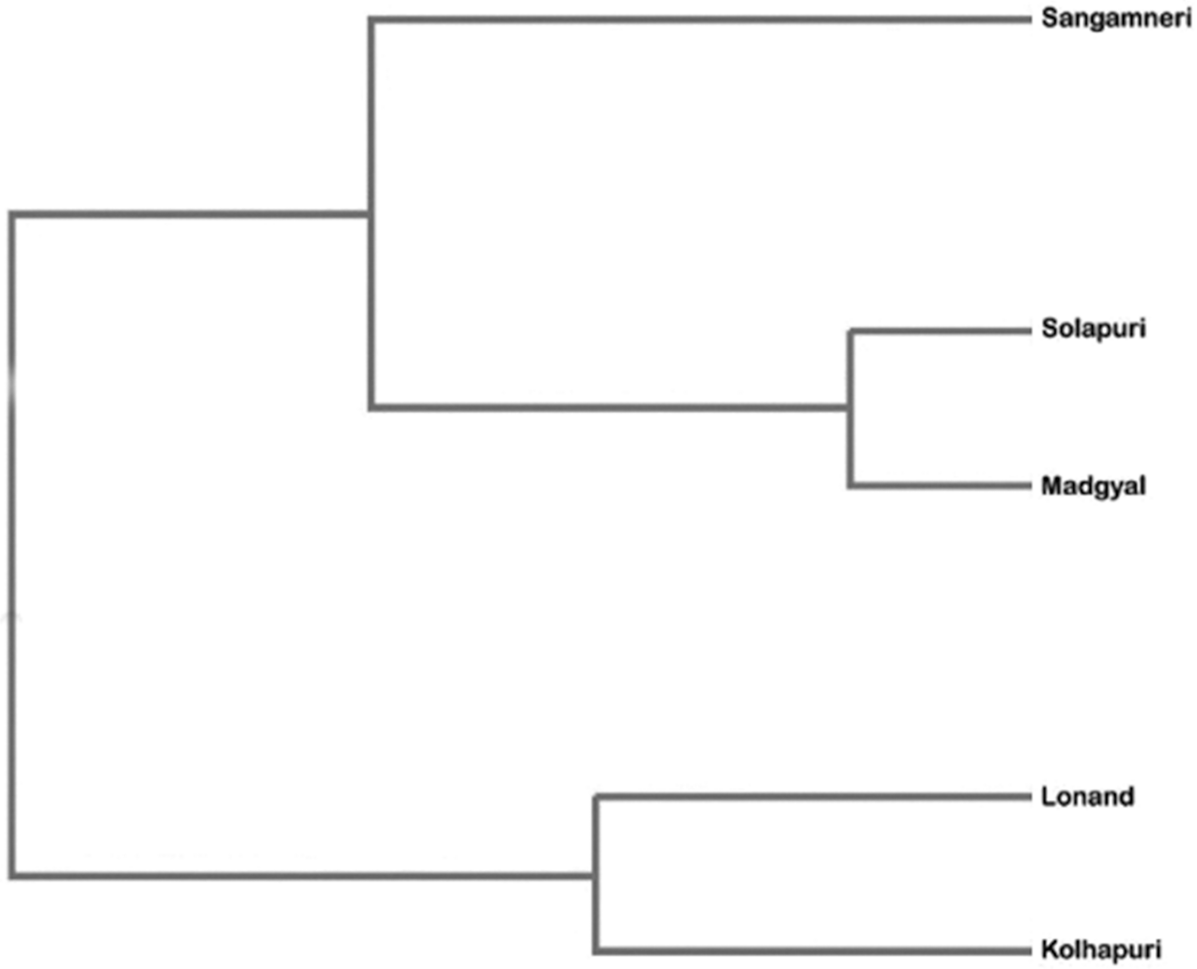
UPGMA based dendrogram using pair-wise Mahalanobis distances.

The UPGMA based dendrogram (Fig 2), constructed on the basis of Mahalanobis distances between the ecotypes, showed two main clusters. One formed by the Sangamneri ecotype as a large group and two sub-clusters of the Madgyal and Solapuri ecotypes. Cluster two included the Lonand and Kolhapuri ecotypes. The discriminant analysis revealed that 352 (31.88%) individuals were misclassified in their source genetic groups. The Sangamneri ecotype showed the least assignment error (22%) whilst the Solapuri ecotype exhibited maximum error level (41%) as depicted by Table 4.

**Table 4:** Percent (%) of individual sheep classified into five genetic groups.

**Ecotype**	Lonand	Solapuri	Madgyal	Kolhapuri	Sangamneri
Lonand	**61**	5	2	21	11
Solapuri	4	**59**	17	4	16
Madgyal	2	19	**66**	0	13
Kolhapuri	15	4	1	**74**	6
Sangamneri	6	4	3	9	**78**
Error level	0.39	0.41	0.34	0.26	0.22
Priors	0.20	0.20	0.20	0.20	0.20

### Microsatellite variation

A total of 407 alleles were found across 25 loci in the 456 individuals sampled from the 5 sheep ecotypes (Table 5). The most polymorphic locus was MAF214 with 25 alleles while BM8125 and OarVH72 were observed to be the least polymorphic with 10 alleles across the 5 sheep ecotypes. A total of 36 population specific (private) alleles were detected but only 7 of them possessed frequencies >5%, of these four were detected in Sangamneri, one in Kolhapuri and two in Madgyal sheep. All the microsatellite markers were highly polymorphic with PIC values ≥0.5 [31] with an average of 0.76. The exact p-values estimated using Markov chain for deviations from Hardy-Weinberg Equilibrium (HWE) are presented in supplementary S3 Table. Deviations from HWE were statistically significant (P<0.05) for 4 loci in Lonand (OarCP49, OarCP20, OarJMP29, OarFCB48), three loci in Madgyal (OarCP49, OarCP20, OarHH41) and one loci each in Sangamneri (OarJMP29), Kolhapuri (OarCP20) and Solapuri (OarCP49) ecotypes. The mean observed and expected heterozygosity across all markers was 0.53 and 0.83 respectively (Table 5).

**Table 5:** Diversity indices, F-statistics (F_IS_, F_IT_, F_ST_) according to Weir and Cockerham (1984), values for 25 microsatellite markers.

Locus	N_a_	N_e_	AR	H_o_	H_e_	PIC	F_IS_	F_IT_	F_ST_
BM757	15	5	6.76	0.59	0.8	0.75	0.255	0.267	0.016
BM827	12	7.13	8.51	0.43	0.86	0.82	0.491	0.498	0.013
BM1314	18	6.9	9.31	0.27	0.86	0.66	0.605	0.696	0.231
BM6506	11	3.62	5.73	0.36	0.72	0.50	0.331	0.537	0.308
BM6526	19	7.16	10.06	0.54	0.86	0.80	0.354	0.379	0.038
BM8125	10	2.88	5.7	0.61	0.65	0.60	0.044	0.071	0.028
CSRD247	23	9.35	11.63	0.39	0.89	0.86	0.551	0.563	0.026
CSSM31	15	9.07	9.7	0.59	0.89	0.86	0.328	0.338	0.016
CSSM47	19	3.75	7.03	0.35	0.73	0.65	0.489	0.534	0.088
HSC	20	9.47	10.6	0.61	0.9	0.87	0.316	0.318	0.004
INRA63	22	8.56	10.25	0.6	0.88	0.85	0.31	0.324	0.021
MAF214	25	3.53	8.01	0.4	0.72	0.65	0.424	0.446	0.038
OarAE129	18	7.83	9.04	0.32	0.87	0.71	0.566	0.649	0.19
OarCP20	14	5.67	7.79	0.89	0.82	0.74	-0.144	-0.064	0.07
OarCP34	13	5.9	8.11	0.45	0.83	0.80	0.452	0.459	0.013
OarCP49	13	8.88	9.63	0.79	0.89	0.80	0.034	0.125	0.095
OarFCB128	22	7.13	9.97	0.53	0.86	0.82	0.034	0.125	0.095
OarFCB48	15	9	10.22	0.62	0.89	0.81	0.252	0.313	0.082
OarHH35	14	4.6	7.85	0.58	0.78	0.74	0.239	0.26	0.027
OarHH41	17	6.48	8.71	0.64	0.85	0.80	0.227	0.243	0.02
OarHH47	15	6.25	9.03	0.64	0.84	0.80	0.232	0.241	0.012
OarHH64	15	5.49	8.22	0.54	0.82	0.78	0.331	0.342	0.017
OarJMP8	14	7.34	9.39	0.52	0.86	0.83	0.394	0.406	0.021
OarJMP29	18	4.43	7.6	0.61	0.78	0.72	0.199	0.216	0.021
OarVH72	10	4.93	7.06	0.47	0.8	0.72	0.389	0.423	0.055
Mean	16.28	6.41	8.71	0.53	0.83	0.76	0.322	0.362	0.059

Na: Observed number of alleles; Ne: Effective number of alleles; AR: Allelic richness; Ho: Observed heterozygosity; He: Expected heterozygosity;

Table 6 summarizes the population-wise allelic patterns across the ecotypes. Considerable neutral genetic variation was observed. Kolhapuri ecotype showed the highest allele diversity (12.52) with a total number of 313 alleles, whereas Sangamneri had the lowest allele diversity (10.92) and a total number of 273 alleles. The observed heterozygosity (H_o_) for all the ecotypes ranged from 0.47 (Sangamneri) to 0.62 (Lonand) whereas the gene diversity values varied from 0.76 (Sangamneri) to 0.81 (Solapuri). All sheep ecotypes were genetically diverse at the 25 loci as evident from the high allele (>6) and gene (>0.6) diversity values. The allelic richness, independent of the sample size revealed highest mean allelic richness in Solapuri (8.25) and least in Sangamneri (7.27) sheep. Marker wise observed and expected heterozygosity in each breed is being given as supplementary information (S4 Table).

**Table 6:** Effective no of alleles (N_a_), allelic richness, expected heterozygosity (H_e_) and observed heterozygosity (H_o_) and within population inbreeding coefficient (F_IS_) across the sheep ecotypes.

Ecotype	Total no of alleles	Effective no of alleles (N_a_)	Allelic richness	Expected Heterozygosity (H_e_)	Observed Heterozygosity (H_o_)	F_IS_
Lonand	292	11.68	7.92	0.78	0.62	0.209
Solapuri	306	12.24	8.25	0.81	0.51	0.360
Madgyal	298	11.92	7.56	0.75	0.51	0.334
Kolhapuri	313	12.52	8.16	0.80	0.56	0.288
Sangamneri	273	10.92	7.27	0.76	0.47	0.379

### Genetic differentiation/relationship

**Fig 3:**
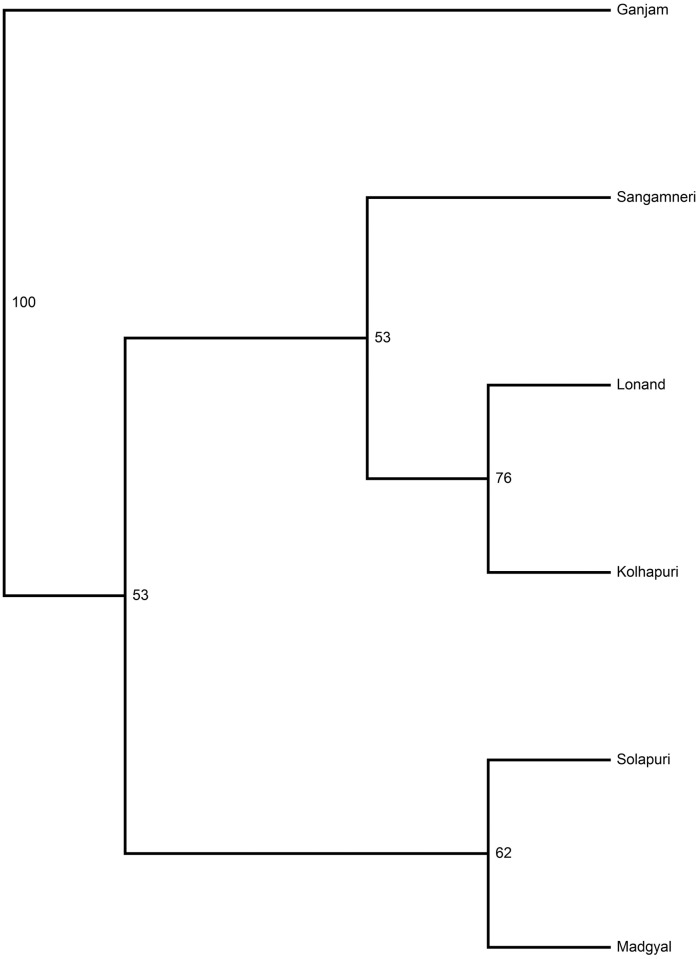
UPGMA tree constructed from Nei’s Minimum (1972) genetic distance depicting relationship of 5 Deccani sheep ecotypes with out-group (Ganjam). Numbers indicate the proportion of bootstrap replicates sharing the labeled node in a bootstrap resampling of 100 replicates.

**Fig 4:**
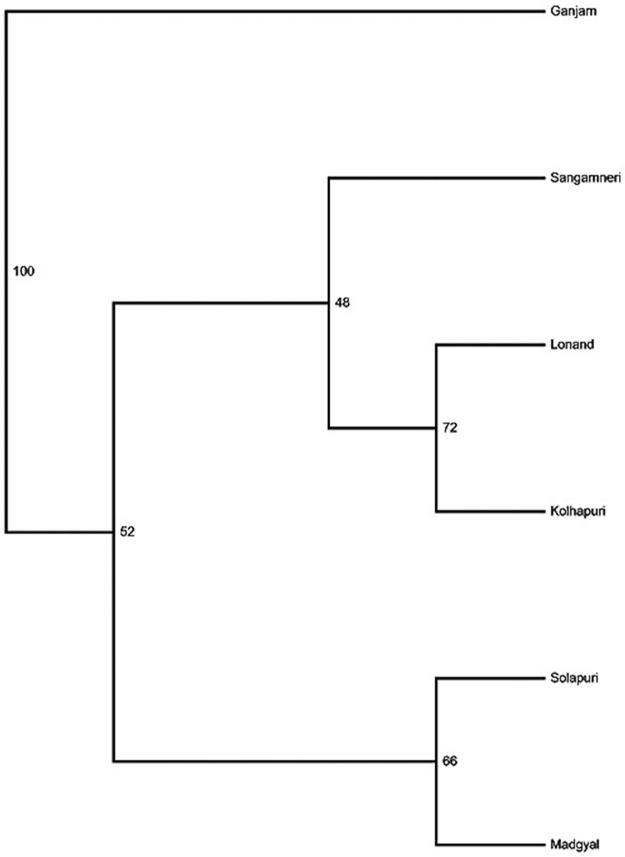
UPGMA tree constructed from Reynold’s (1983) genetic distance depicting relationship of 5 Deccani sheep ecotypes with out-group (Ganjam). Numbers indicate the proportion of bootstrap replicates sharing the labeled node in a bootstrap resampling of 100 replicates.

The global analysis of Wright’s F-statistics revealed a 32.2% deficit of heterozygotes for each of the analysed ecotypes whereas the total population had a 36.2% deficit of heterozygotes (Table 5). The interbreed population differentiation (F_ST_) and genetic distance (D_S_) matrices are given in Table 7. All the F_ST_ values between populations were statistically significant (p<0.05).The ecotype pair-wise F_ST_ values ranged from 0.025 to 0.054, thereby revealing least differentiation between Solapuri and Madgyal and highest differentiation between Sangamneri and Madgyal. The average genetic differentiation (F_ST_) between the ecotypes was 5.9% (P<0.05) revealing moderate discrimination between the ecotypes (Table 5). In terms of genetic distances, Kolhapuri and Lonand were most closely related (Ds = 0.177) while Sangamneri and Madgyal appeared distinct (Ds = 0.390).) Fig 3 shows the UPGMA tree obtained from Nei’s distance measures (1972) between the ecotypes. High bootstrap values (>50%) were obtained for Solapuri-Madgyal and Lonand -Kolhapuri, while Sangamneri was distinct. Reynolds (1983) genetic distance was also used to estimate the genetic relationship between the ecotypes, using Ganjam sheep breed from eastern region as out-group (Fig 4). The results were in concordance with F_ST_ and Ds, with Sangamneri as distinct ecotype and a close relationship of Lonand –Kolhapuri and Solapuri-Madgyal, with high bootstrap values.

**Table 7:** F_ST_ Values below diagonal and Nei unbiased genetic distance (D_s_) above the diagonal.

	Lonand	Sangamneri	Kolhapuri	Solapuri	Madgyal
Lonand	0.000	0.228	0.177	0.285	0.363
Sangamneri	0.032	0.000	0.342	0.338	0.390
Kolhapuri	0.026	0.043	0.000	0.243	0.229
Solapuri	0.034	0.041	0.028	0.000	0.153
Madgyal	0.049	0.054	0.032	0.025	0.000

**Fig 5:**
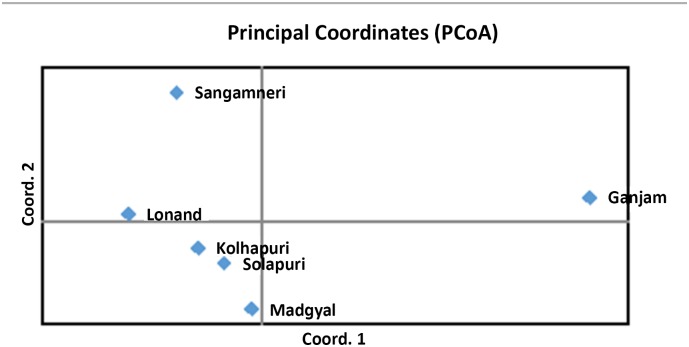
PCA plot for the ecotypes.

In a factorial correspondence analysis, the first three components accounted for 80.05%, 8.07% and 6.02% of the total variation respectively. The PCA plot for the breeds (Fig 5) revealed Ganjam, the out-group as a distinct breed while Sangamneri is separated from rest of the ecotypes. The results of the Principal Component analysis are in agreement with the phylogenetic tree obtained in the present study.

Bayesian cluster analysis on the entire data set clustered the ecotypes into 5 groups.

The data was also used for population assignment [39]. A total of 88% animals were correctly assigned to their source population. Highest assignment or purity was observed in Sangamneri (94%) and least in Lonand (83.6%) (Table 8).

**Table 8:** Summary of population assignment [39].

Population	Self-Population	Other-Population
Lonand	51	10
Sangamneri	94	6
Kolhapuri	83	16
Solapuri	87	11
Madgyal	86	12
Total	401	55
%	88	12

**Fig 6:**
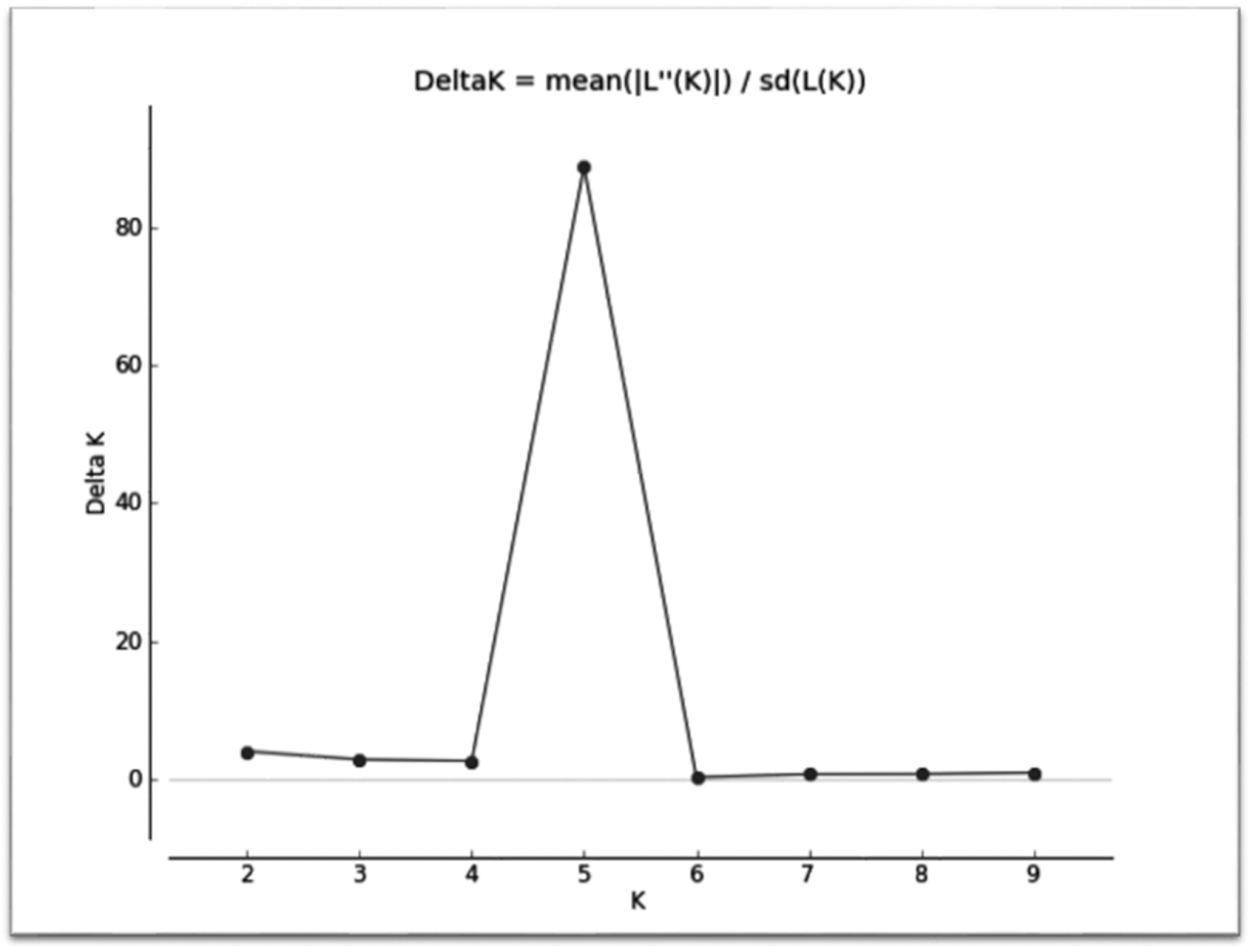
Graph of delta K values to determine the ideal number of groups present in 5 sheep ecotypes. The rate of change in the log-likelihood values between the number of genetic populations, K, for K = 2 to K = 9, showing that the value of K with the greatest support is K = 5.

**Fig 7:**
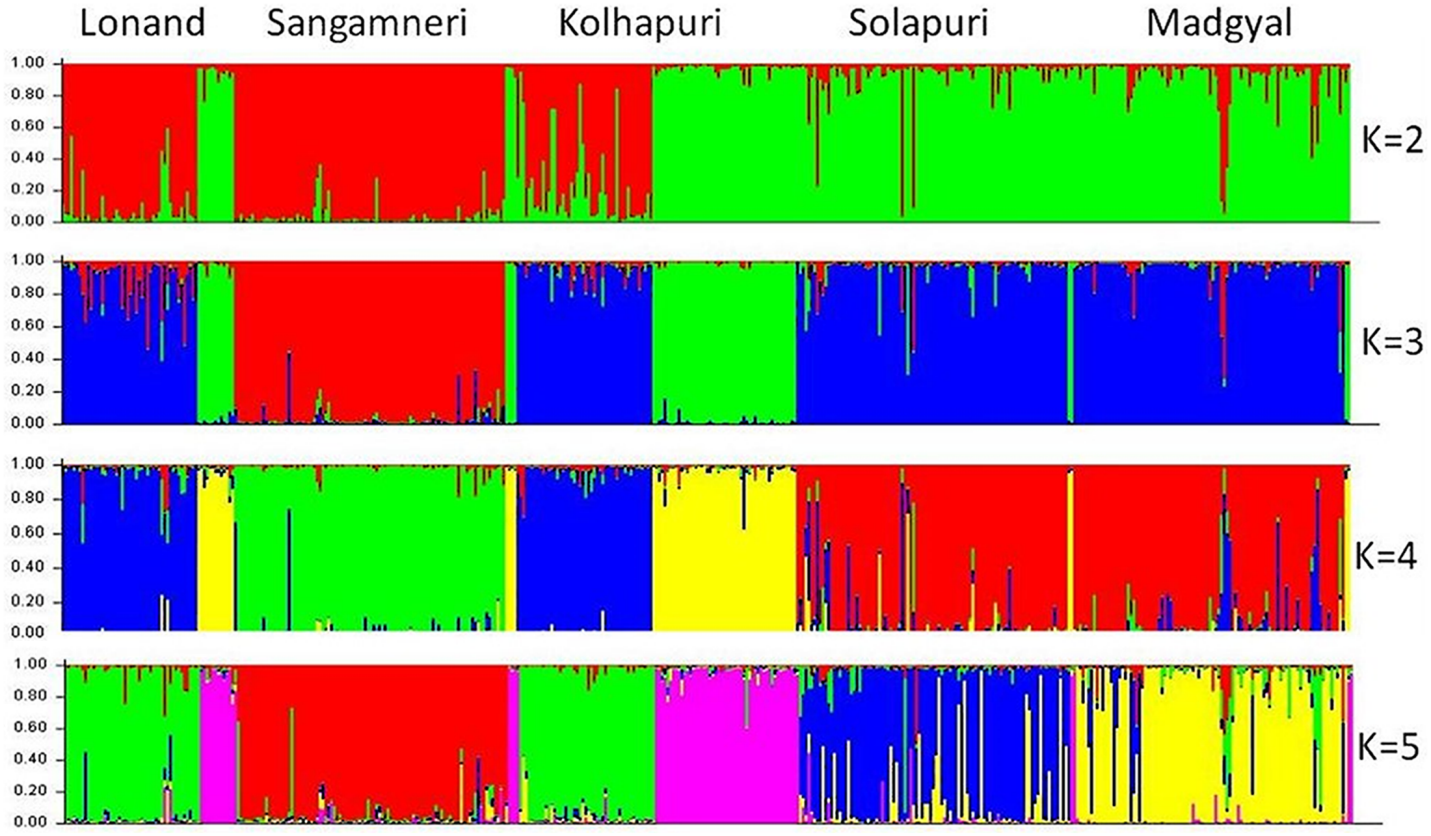
Clusters inferred from STRUCTURE at K = 2–5. The cluster membership of each sample is shown by the colour composition of the vertical lines, with the length of each colour being proportional to the estimated membership coefficient.

These clusters were inferred only on the basis of allele frequency differences. As shown in Fig 6, the likelihood of the observed data increases steadily when the number of clusters increases and tends to reach an asymptote. The average membership coefficient (q) for each ecotype for K = 5 is depicted in Table 9. At K = 5, Lonand, Sangamneri, Solapuri and Madgyal animals form their own clusters (q>0.7), whereas Kolhapuri sheep cluster together with Lonand (q = 0.456) and also form a separate cluster (q = 0.493) (Fig 7).

**Table 9:** Proportion of membership of each of the 5 analysed sheep breeds/populations in the 5 inferred clusters derived using STRUCTURE software.

	1	2	3	4	5
Lonand	**0.719**	0.208	0.033	0.014	0.025
Solapuri	0.039	0.042	0.019	0.135	**0.765**
Madgyal	0.062	0.031	0.037	**0.776**	0.094
Kolhapuri	**0.456**	**0.493**	0.013	0.023	0.015
Sangamneri	0.031	0.052	**0.884**	0.018	0.016

### Conservation priority

The Weitzman marginal loss of diversity attached to each ecotype is shown in Table 10. The contribution of each ecotype to overall diversity ranged from 16.8% to 26.6%. The results of threat status and breed merits are shown in Tables 11 and 12. Extinction probability was highest in Lonand sheep (0.9) and least in Madgyal sheep (0.3). Socio-cultural score ranged from 0.33 to 0.35. Economic merit score ranged from 0.1 (Lonand) to 0.4 (Madgyal) depicting highest economic merit of the Madgyal sheep. The economic gains from Sangamneri and Kolhapuri sheep were similar. Conservation priority of five sheep ecotypes based on their total utility is presented in Table 13. The highest conservation priority ecotype was Lonand followed by Kolhapuri. The study gave least conservation priority to Solapuri sheep.

**Table 10:** Total and marginal diversity of the ecotypes (Weitzman).

	V(S-i)	dV(i)	dV(i) %	V(S)
Lonand	0.844	0.218	20.5	1.062
Solapuri	0.884	0.178	16.8	
Madgyal	0.851	0.211	19.9	
Kolhapuri	0.852	0.210	19.8	
Sangamneri	0.780	0.282	26.6	

V(S)—diversity of set; V(S-i)—diversities of set without element i; dV(i)—marginal diversity; dV(i) %-marginal diversity in percent

**Table 11:** Ecotype threat score.

*Ecotype*	Threat Score				
	Population Size	Average number of rams per flock	Level of crossbreeding	Maintenance of pure stock	Farmers opinion towards the ecotype
Lonand	32600	0.30	3.8	0.8	2.20
Solapuri	36935	0.86	2.0	1.0	2.48
Madgyal	48874	1.20	0.2	1.8	2.98
Kolhapuri	45230	1.60	1.0	1.3	2.30
Sangamneri	51200	0.98	1.2	1.3	2.64

**Table 12:** Ecotype merit score.

Ecotype	Merit Score			
	Economic	Ecological	Cultural	Average
Lonand	0.1	0.2	0.35	0.22
Solapuri	0.2	0.2	0.34	0.25
Madgyal	0.4	0.3	0.33	0.34
Kolhapuri	0.3	0.3	0.35	0.32
Sangamneri	0.3	0.3	0.34	0.31

**Table 13:** Conservation priorities based on Weitzman diversity, extinction probability and current ecotype merits.

Ecotype	Contribution to diversity (Weitzman)	Extinction probability	Average ecotype merit	Total utility	Conservation priority
Lonand	0.218	0.90	0.22	0.612	1
Solapuri	0.178	0.55	0.25	0.446	5
Madgyal	0.211	0.30	0.34	0.467	4
Kolhapuri	0.210	0.45	0.32	0.509	2
Sangamneri	0.282	0.35	0.31	0.507	3

## Discussion

### Morphological diversity

The average values of BW, BL, WH and CG in Madgyal sheep were comparable to those in Munjal sheep and the corresponding values in Solapuri were similar to Muzaffarnagri sheep [15]. The averages for BW, BL, WH and CG in Kolhapuri sheep were similar to those reported for Bellary sheep [12]. Moreover, morphometric traits increased with the age, reflecting the body growth with age. The same tendency was reported by Yadav et al in other Indian sheep breeds [45]. Also, there was a certain sexual dimorphism (morphometric traits of males had the higher values than females) in all studied traits, except in EL. Similar results were reported in Djallonke and Sahel sheep in Northern Ghana [46] and four ovine breeds of southern peninsular zone of India [12]. The most discriminating traits between the studied ecotypes were TL followed by HW, BL, EL, BW, PG and CG. Similar discriminating factors were reported in indigenous Nigerian and Indian sheep [11–12, 45].

Based on morphometric measurements, the large distances between Madgyal and other ecotypes (Kolhapuri, Lonand and Sangamneri) might be due to its development under selective breeding. Madgyal is an improved sheep whose origins are obscure [47]. Some shepherds say they are the result of the selective breeding around 100 years ago by local sheep breeders at Madgyal and the adjoining village Sanmadi. Maintenance of pure elite rams and ewes to produce breeding stock for sale at best price is the unique feature of sheep husbandry in the Madgyal sheep breeding tract [48]. The smallest distance between Madgyal and Solapuri ecotypes may be due to small differences in their body size. The dendrogram (Fig 2) based on Mahalanobis distances was similar to the formation of two large groups reported in Andalusian caprine breeds [49]. The relationship of the ecotypes in the dendrogram could be attributed to the influence of different productive ability and different breed origins [49]. This might be partly attributed to differences in management practices, agro-climatic conditions and biophysical resources [12]. Herrera [49] indicated that head profile was most important in determining different racial origin of the breeds. Madgyal and Solapuri sheep have a typical roman nose as judged against Lonand, Sangamneri and Kolhapuri ecotypes [25]. Thus, Madgyal is closely related to the Solapuri, and Lonand is closely related to Kolhapuri ecotype. The Sangamneri ecotype appears to be far from these four populations. Also, fewer Sangamneri animals were erroneously assigned, indicating homogeneity and distinctiveness of Sangamneri ecotype. A similar result was found between Florida and Andalusia goat populations [49].

### Genetic diversity

The allele diversity based on mean number of alleles and mean effective allele number revealed high genetic polymorphism in the investigated sheep populations. The genetic diversity is also reflected in the phenotypic variability between these ecotypes. This magnitude of genetic diversity can be attributed to the highly polymorphic microsatellite markers used (PIC>0.5). These estimates are higher than those reported for other Indian sheep breeds [13, 17, 51] which may be reflective of large sample size used in this study. A higher genetic diversity has been reported in Turkish sheep breeds, which is probably indicative of the area as a centre of sheep domestication [50]. Allelic richness in our study was comparable to that reported for Ethiopian, European and Nigerian sheep breeds [8, 52–54]. The observed heterozygosity values of the five ecotypes were relatively similar to those of other domestic sheep breeds reported earlier [13, 18, 51]. The gene diversity values across the ecotypes in the present study were comparable to Turkish sheep breeds [50] but higher than other Indian [13, 18, 51], Spanish Assaf [14], Algerian [55] and Moroccan sheep breeds [56]. Since a different set of markers has been used in these studies, the comparisons can only be indicative. The high value of gene diversity indicated that the ecotypes had retained the presence of several alleles although at low frequencies. This implied a substantial amount of genetic variability in them that might be used in planning breeding strategies particularly in populations of small sizes.

A significant positive *F*_IS_ value revealed heterozygotes deficiency in the five ecotypes. Possible reasons could be presence of null alleles; locus may be under selection; or population subdivision. However, distinguishing among these is generally difficult. Due to non-availability of pedigreed animals with the farmers for analysis, it was not possible to demonstrate the presence of null alleles. Inbreeding is an unlikely explanation because of the presence of high gene diversity in the populations and observing the practice of replacing the breeding rams in the farmer’s sheep flocks. The deficit of heterozygotes may partly be explained by Wahlund effects at our level of sampling i.e. sampling at random from several flocks in several villages [57]. Further, the relatedness of few samples otherwise deemed unrelated during collection may not be denied due to non-availability of pedigreed data under field conditions. Since the population status of the sheep ecotypes investigated is not known, a bottleneck analysis was done to investigate the effects of temporal changes on the genetic diversity. Efforts made to study recent bottleneck effect (up to 40–80 generations) in the studied sheep ecotypes by using the Mode shift test revealed normal L-shaped curves. This finding clearly suggested the absence of a recent reduction in the effective population size or a genetic bottleneck. [58]. Similar results have been reported for other Indian sheep breeds [59].

The population differences shown by global analysis of F_ST_ (coefficient of multi-locus genetic differentiation fixation index) were moderate (Wright, 1978) with 5.9% of total genetic variation attributable to breed differences and the remaining 94.1% corresponding to differences among individuals. This could be due to geographic proximity, migration and cultural similarities amongst the major sheep rearing communities. Similar reasons were attributed to low differentiation (4.6%) between the Ethiopian sheep breeds [8]. The observed differentiation (5.9%) is lesser to the differences observed in sheep breeds/populations from North-western semi-arid zone (7.9%), and Southern peninsular and Eastern regions (13.2%) of India [13, 18]. The authors attributed lack of strict breeding rules and geographical isolation between the investigated Indian sheep breeds as the reasons to the moderate level of differentiation among them. Low to moderate population structure were reported in Turkish (10.8%; [50]), Alpine (5.7%; [52], Nigerian (8.8%; [53], Algerian (3.8%; [55], Moroccan (3.6%; [56]) and Austrian (8%; [60] sheep breeds.

The frequency-based assignment test [39] assigns a sample to a population with the highest log likelihood value. Sangamneri ecotype had the highest percentage (94%) of correctly assigned individuals while Lonand had the least (83.6%). These results are supported by discriminant analysis of morphometric data of Deccani sheep ecotypes.Analysis of genetic relationship and differentiation between the ecotypes based on F_ST_, genetic distances and PCA revealed similar results, with Sangamneri ecotype differentiated from other sheep in the study. Genetic closeness was observed between Lonand and Kolhapuri as well as Solapuri and Madgyal. The phylogenetic relationship between the ecotypes was supported by the cluster analyses. The clusters defined by STRUCTURE [40] and by the model value of the distribution of the ΔK [61] showed clustering of individuals in five groups indicating a genetic subdivision within the Deccani sheep breed. The five clusters are consistent with the observed morphological classification. Evaluation of the clusters revealed the presence of admixtures which are indicative of gene flow between these ecotypes. Sangamneri is the only ecotype with least admixture. This may be due to geographical delineation of the ecotype and the management practices of the sheep owners. The farmers followed traditional breeding systems under extensive management. Uncontrolled breeding with and without true to breed (pure) rams was observed (average number of rams and average number of pure rams per flock in Lonand ecotype were 1.6 and 0.3 respectively. Similarly, the corresponding values in Kolhapuri ecotype were 2.3 and 1.6). The genetic distances held the close relationship of Solapuri and Madgyal and the highest distance between Sangamneri and Madgyal sheep. The clustering based on microsatellite data in our study is in congruence with the clustering based on morphometric data.

### Conservation priority

The measure of the marginal loss of diversity for each ecotype using the Weitzman approach enables the ranking of breeds for conservation purposes [62]. This approach was used for estimating the contribution of each ecotype to the total diversity of the breeds, in order to clarify the relative importance of individual ecotypes investigated. The relative loss of genetic diversity caused by loss of one specific breed/population, can be regarded as a measure of uniqueness of individual breeds in comparison to the complete set and is quantified by the Weitzman approach [62]. In the investigated set of ecotypes, the highest loss of diversity would be incurred with removal of Sangamneri (26.6%) and the lowest from the removal of Solapuri (16.8%) sheep. Sangamneri sheep depicting a higher marginal diversity (>26%) were potential contenders for conservation precedence over other investigated ecotypes. Based on genetic and non-genetic indicators, our study ranked five indigenous sheep ecotypes of Maharashtra for conservation by combining threat indicators, current breed merits and contribution to genetic diversity. Earlier studies, except [21, 42, 44] have set conservation priorities of livestock breeds based solely on genetic diversity indicators. [44], in a similar study on Ethiopian sheep, reported that the relative conservation priorities of the sheep breeds changed when they were ranked based on their contribution to genetic diversity or on their total utility. Our results (Table 13) are in agreement to those reported by [44]. The results show that the conservation priorities differ if we use only genetic diversity indicators for ranking the breeds. Weitzman diversity approach gives highest priority to the conservation of Sangamneri sheep whereas the maximum-utility approach gives highest priory to Lonand sheep.

## Conclusion

The study defines the morphometric and genetic standards of Deccani sheep ecotypes. Morphometric as well as genetic differentiation established five differentiated groups. A fair congruence between the dendrogram constructed on the basis of Mahalanobis distances and Nei’s genetic distances was observed. The gene diversity observed was moderate, a distinctive trait of India sheep breeds. It may notably be the result of the lack of artificial selection pressure and high level of genetic admixture in the breeds. Our study outlines conservation priorities of Deccani sheep ecotypes by combining contributions to genetic diversity, breed merits and threat status. The study could be used as a model to define conservation priorities of Indian sheep breeds and could contribute to a National conservation plan of Indian livestock breeds.

## Supporting information

S1 Table Number of animals used in the study. Here is the ecotype-wise information about the total number of animals used for morphometric characterization and for genotyping.
(DOCX)

S2 Table Primer sequences type of repeat, size range, location and accession numbers. Here is the information on these parameters on the 25 microsatellites used in the study.
(DOCX)

S3 Table Marker wise observed and expected heterozygosity. This table provides observed and expected heterozygosity in each ecotype on used 25 microsatellite markers.
(DOCX)

S4 Table Exact P-Values by the Markov chain method. The exact p-values using Markov chain (dememorization 10000, batches 20, iteration per batch 5000) were estimated using GENEPOP [32] version 3.1. 1.
(DOCX)

S1 File Allele frequency data of five sheep ecotypes. This file contains the allele frequency data on 456 animals genotyped on 25 microsatellite markers. The animals belong to five sheep ecotypes of Maharashtra, India.
(XLSX)
