# Supplementary material for: Classification and conservation priority of five Deccani sheep ecotypes of Maharashtra, India
Source: PLoS One. 2017 Sep 14;12(9):e0184691. doi: 10.1371/journal.pone.0184691 (PMC5598990; doi:10.1371/journal.pone.0184691)
Supplement: S1 Table — Here is the ecotype-wise information about the total number of animals used for morphometric characterization and for genotyping. (DOCX) [file pone.0184691.s001.docx]

**S1 Table. Ecotype wise no of animals used for morphometric and genetic characterization**

| **Ecotype** | **Morphometric Characterization** | **Genotyping** |
| --- | --- | --- |
| Lonand | 114 | 61 |
| Sangamneri | 184 | 100 |
| Madgyal | 255 | 98 |
| Kolhapuri | 313 | 99 |
| Solapuri | 235 | 98 |
| Total | 1101 | 456 |
