# Supplementary material for: Classification and conservation priority of five Deccani sheep ecotypes of Maharashtra, India
Source: PLoS One. 2017 Sep 14;12(9):e0184691. doi: 10.1371/journal.pone.0184691 (PMC5598990; doi:10.1371/journal.pone.0184691)
Supplement: S2 Table — Here is the information on these parameters on the 25 microsatellites used in the study. (DOCX) [file pone.0184691.s002.docx]

**Table S2. Primer sequences type of repeat, size range, location and accession numbers of the used microsatellites.**

| **Locus** | **Primer sequence** | **Type of repeat** | **Size range(bp)** | **Chr. no.** | **GenBank Acc. no.** |
| --- | --- | --- | --- | --- | --- |
| BM757 | tgg aaa caa tgt aaa cct ggg  ttg agc cac caa gga acc | (GT)_17_ | 178-198 | 9 | G18473 |
| BM827 | ggg ctg gtc gta tgc tga g  gtt gga ctt gct gaa gtg acc | - | 214-224 | 3 | U06763 |
| BM1314 | ttc ctc ctc ttc tct cca aac  atc tca aac gcc agt gtg g | - | 141-161 | 22 | G18433 |
| BM6506 | gca cgt ggt aaa gag atg gc  agc aac ttg agc atg gca c | - | 189-199 | 1 | G18455 |
| BM6526 | cat gcc aaa caa tat cca gc  tga agg tag aga gca agc agc | - | 140-170 | 26 | G18454 |
| BM8125 | ctc tat ctg tgg aaa agg tgg g  ggg ggt tag act tca aca tac g | - | 105-121 | 17 | G18475 |
| CSRD247 | gga ctt gcc aga act ctg caa t  cac tgt ggt ttg tat tag tca gg | (AC)_n_ | 203-237 | 14 | EU009450 |
| CSSM31 | cca agt tta gta ctt gta agt aga  gac tct cta gca ctt tat ctg tgt | AAAA(CA)_7_TA(CA)_25_ | 162-182 | 23 | U03838 |
| CSSM47 | tct ctg tct cta tca cta tat ggc  ctg ggc acc tga aac tat cat cat | (TG)_12_TATGTA(TG)_4_ | 120-160 | 2 | U03821 |
| HSC | ctg cca atg cag aga cac aag a  gtc tgt ctc ctg tct tgt cat c | - | 267-285 | 20 | M90759 |
| INRA63 | gac cac aaa ggg att tgc aca agc  aaa cca cag aaa tgc ttg gaa g | (AC)_13_ | 165-203 | 14 | X71507 |
| MAF214 | aat gca gga gat ctg agg cag gga cg  ggg tga tct tag gga ggt ttt gga gg | - | 187-231 | 16 | M88160 |
| OarAE129 | aat cca gtg tgt gaa aga cta atc cag  gta gat caa gat ata gaa tat ttt tca aca cc | (AC)_14_ | 141-169 | 5 | L11051 |
| OarCP20 | gat ccc ctg gag gag gaa acg g  ggc att tca tgg ctt tag cag g | (AC)_14_ | 67-79 | 21 | U15695 |
| OarCP34 | gct gaa caa tgt gat atg ttc agg  ggg aca ata ctg tct tag atg ctg c | (AC)_17_TTGCGTGT(CA)_4_ | 108-122 | 3 | U15699 |
| OarCP49 | cag aca cgg ctt agc aac taa acg c  gtg ggg atg aat att cct tca taa gg | (AC)_17_ | 80-110 | 17 | U15702 |
| OarFCB48 | gag tta gta caa gga tga caa gag gca c  gac tct aga gga tcg caa aga acc ag | (TG)_11_CA(TG)_3_ | 142-164 | 17 | M82875 |
| OarFCB128 | cag ctg agc aac taa gac ata cat gcg  att aaa gca tct tct ctt tat ttc ctc gc | (GT)_6_GC(GT)_15_ | 97-123 | 2 | L01532 |
| OarHH35 | aat tgc att cag tat ctt taa cat ctg gc  atg aaa ata taa aga gaa tga acc aca cgg | (TG)_17_ | 111-139 | 4 | L12554 |
| OarHH41 | tcc aca ggc tta aat cta tat agc aac c  cca gct aaa gat aaa aga tga tgt ggg ag | (AC)_23_ | 118-140 | 10 | L12555 |
| OarHH47 | ttt att gac aaa ctc tct tcc taa ctc cac c  gta gtt att taa aaa aat atc ata cct ctt aag g | (AC)_32_ | 124-146 | 18 | L12557 |
| OarHH64 | cgt tcc ctc act atg gaa agt tat ata tgc  cac tct att gta aga att tga atg aga gc | (TG)_17_ | 120-134 | 4 | L12558 |
| OarJMP8 | cgg gat gat ctt ctg tcc aaa tat gc  cat ttg ctt tgg ctt cag aac cag ag | (gT)_n_ | 115-129 | 6 | U35059 |
| OarJMP29 | gta tac acg tgg aca ccg ctt tgt ac  gaa gtg gca aga ttc aga ggg gaa g | (CA)_21_ | 86-144 | 24 | U30893 |
| OarVH72 | ctc tag agg atc tgg aat gca aag ctc  ggc ctc tca agg ggc aag agc agg | (AC)14 | 121-133 | 25 | L12548 |
