# Supplementary material for: Classification and conservation priority of five Deccani sheep ecotypes of Maharashtra, India
Source: PLoS One. 2017 Sep 14;12(9):e0184691. doi: 10.1371/journal.pone.0184691 (PMC5598990; doi:10.1371/journal.pone.0184691)
Supplement: S3 Table — This table provides observed and expected heterozygosity in each ecotype on used 25 microsatellite markers. (DOCX) [file pone.0184691.s003.docx]

**S3 Table. Marker wise observed and expected heterozygosity in each breed.**

| **Locus** | **Lonand** | | **Solapuri** | | **Madgyal** | | **Kolhapuri** | | **Sangamneri** | |
| --- | --- | --- | --- | --- | --- | --- | --- | --- | --- | --- |
|  | **Ho** | **He** | **H_o_** | **H_e_** | **H_o_** | **H_e_** | **H_o_** | **H_e_** | **Ho** | **He** |
| BM0757 | 0.436 | 0.782 | 0.615 | 0.805 | 0.600 | 0.784 | 0.587 | 0.743 | 0.655 | 0.801 |
| BM0827 | 0.449 | 0.830 | 0.310 | 0.857 | 0.403 | 0.873 | 0.654 | 0.857 | 0.385 | 0.773 |
| BM1314 | 0.492 | 0.711 | 0.207 | 0.863 | 0.433 | 0.535 | 0.313 | 0.817 | 0.010 | 0.513 |
| BM6506 | 0.448 | 0.520 | 0.573 | 0.772 | 0.161 | 0.466 | 0.341 | 0.471 | 0.257 | 0.421 |
| BM6526 | 0.689 | 0.813 | 0.420 | 0.868 | 0.542 | 0.927 | 0.517 | 0.785 | 0.573 | 0.732 |
| BM8125 | 0.586 | 0.680 | 0.859 | 0.648 | 0.533 | 0.605 | 0.561 | 0.616 | 0.540 | 0.643 |
| CSRD247 | 0.611 | 0.831 | 0.357 | 0.869 | 0.526 | 0.814 | 0.283 | 0.910 | 0.100 | 0.928 |
| CSSM31 | 0.316 | 0.878 | 0.651 | 0.883 | 0.520 | 0.848 | 0.557 | 0.906 | 0.742 | 0.856 |
| CSSM47 | 0.518 | 0.769 | 0.298 | 0.636 | 0.247 | 0.462 | 0.493 | 0.832 | 0.290 | 0.756 |
| HSC | 0.500 | 0.851 | 0.493 | 0.911 | 0.731 | 0.878 | 0.500 | 0.898 | 0.738 | 0.862 |
| INRA63 | 0.849 | 0.878 | 0.306 | 0.908 | 0.565 | 0.838 | 0.625 | 0.856 | 0.716 | 0.843 |
| MAF214 | 0.482 | 0.651 | 0.239 | 0.862 | 0.386 | 0.574 | 0.535 | 0.762 | 0.385 | 0.615 |
| OarAE129 | 0.455 | 0.769 | 0.230 | 0.800 | 0.143 | 0.500 | 0.402 | 0.799 | 0.402 | 0.797 |
| OarCP20 | 0.984 | 0.760 | 0.878 | 0.796 | 0.918 | 0.791 | 0.919 | 0.764 | 0.786 | 0.755 |
| OarCP34 | 0.464 | 0.866 | 0.530 | 0.773 | 0.550 | 0.786 | 0.462 | 0.854 | 0.214 | 0.840 |
| OarCP49 | 0.828 | 0.849 | 0.924 | 0.736 | 0.969 | 0.874 | 0.649 | 0.823 | 0.611 | 0.804 |
| OarFCB128 | 0.630 | 0.866 | 0.458 | 0.798 | 0.582 | 0.772 | 0.580 | 0.879 | 0.154 | 0.891 |
| OarFCB48 | 0.845 | 0.883 | 0.523 | 0.855 | 0.456 | 0.880 | 0.640 | 0.865 | 0.677 | 0.680 |
| OarHH35 | 0.759 | 0.852 | 0.629 | 0.740 | 0.472 | 0.602 | 0.607 | 0.849 | 0.470 | 0.775 |
| OarHH41 | 0.750 | 0.779 | 0.723 | 0.853 | 0.853 | 0.822 | 0.598 | 0.825 | 0.260 | 0.840 |
| OarHH47 | 0.778 | 0.818 | 0.692 | 0.767 | 0.471 | 0.878 | 0.671 | 0.804 | 0.658 | 0.844 |
| OarHH64 | 0.550 | 0.750 | 0.636 | 0.866 | 0.254 | 0.861 | 0.632 | 0.769 | 0.561 | 0.770 |
| OarJMP08 | 0.727 | 0.810 | 0.352 | 0.900 | 0.273 | 0.868 | 0.577 | 0.819 | 0.663 | 0.820 |
| OarJMP29 | 0.673 | 0.707 | 0.410 | 0.790 | 0.652 | 0.822 | 0.721 | 0.771 | 0.612 | 0.674 |
| OarVH72 | 0.593 | 0.690 | 0.438 | 0.786 | 0.415 | 0.763 | 0.592 | 0.666 | 0.338 | 0.857 |
