# Supplementary material for: Classification and conservation priority of five Deccani sheep ecotypes of Maharashtra, India
Source: PLoS One. 2017 Sep 14;12(9):e0184691. doi: 10.1371/journal.pone.0184691 (PMC5598990; doi:10.1371/journal.pone.0184691)
Supplement: S4 Table — The exact p-values using Markov chain (dememorization 10000, batches 20, iteration per batch 5000) were estimated using GENEPOP [32] version 3.1. 1. (DOCX) [file pone.0184691.s004.docx]

**S4 Table. Estimation of exact P-Values by the Markov chain method**

| **Locus** | **Lonand** | **Sangamneri** | **Kolhapuri** | **Solapuri** | **Madgyal** |
| --- | --- | --- | --- | --- | --- |
| BM0757 | 0.000 | 0.000 | 0.001 | 0.000 | 0.000 |
| BM8125 | 0.009 | 0.000 | 0.006 | 0.001 | 0.000 |
| BM0827 | 0.000 | 0.000 | 0.001 | 0.000 | 0.000 |
| OarCP49 | **0.231** | 0.000 | 0.000 | **0.231** | **0.999** |
| OarHH47 | 0.009 | 0.000 | 0.000 | 0.071 | 0.000 |
| CSSM47 | 0.000 | 0.000 | 0.000 | 0.000 | 0.000 |
| MAF214 | 0.002 | 0.000 | 0.000 | 0.000 | 0.000 |
| OarCP20 | **1.000** | 0.000 | **1.000** | 0.022 | **0.052** |
| OarHH41 | 0.004 | 0.000 | 0.000 | 0.000 | **0.693** |
| OarVH72 | 0.006 | 0.000 | 0.007 | 0.000 | 0.000 |
| BM6526 | 0.000 | 0.008 | 0.000 | 0.000 | 0.000 |
| INRA63 | 0.044 | 0.000 | 0.000 | 0.000 | 0.000 |
| OarAE129 | 0.000 | 0.000 | 0.000 | 0.000 | 0.000 |
| OarCP34 | 0.000 | 0.000 | 0.000 | 0.000 | 0.000 |
| OarFCB128 | 0.000 | 0.000 | 0.000 | 0.000 | 0.000 |
| HSC | 0.000 | 0.011 | 0.000 | 0.000 | 0.000 |
| OarHH35 | 0.047 | 0.000 | 0.000 | 0.000 | 0.001 |
| OarHH64 | 0.000 | 0.000 | 0.000 | 0.000 | 0.000 |
| OarJMP29 | **0.565** | **0.201** | 0.031 | 0.000 | 0.000 |
| OarJMP08 | 0.004 | 0.000 | 0.000 | 0.000 | 0.000 |
| BM1314 | 0.000 | 0.000 | 0.000 | 0.000 | 0.000 |
| BM6506 | 0.000 | 0.000 | 0.000 | 0.000 | 0.000 |
| CSRD247 | 0.000 | 0.000 | 0.000 | 0.000 | 0.000 |
| OarFCB48 | **0.135** | 0.027 | 0.000 | 0.000 | 0.000 |
| CSSM31 | 0.000 | 0.001 | 0.000 | 0.000 | 0.000 |
